# Inflammatory factors in alveolar lavage fluid from severe COVID-19 pneumonia: PCT and IL-6 in epithelial lining fluid

**DOI:** 10.1515/med-2021-0330

**Published:** 2021-08-09

**Authors:** Franz J. Wiedermann, Wolfgang Lederer

**Affiliations:** Department of Anaesthesiology and Critical Care Medicine, Medical University of Innsbruck, Anichstrasse 35, 6020 Innsbruck, Austria; Department of Anaesthesiology and Critical Care Medicine, Medical University of Innsbruck, Austria

Dear Editor,

The research article “Clinical Significance of Cellular Immunity and Inflammatory Factors Assays in Alveolar Lavage Fluid for Severe COVID-19 Pneumonia” by Liao and Yang [1] reported that the concentrations of interleukin-6 (IL-6), C-reactive protein (CRP), and procalcitonin (PCT) in the alveolar lavage fluid and in the serum of survivors were lower compared to those of non-survivors. Levels of IL-6, CRP, and PCT were higher in samples of alveolar lavage than in the serum, indicating that viral infections lead to abnormal oxygen and carbon dioxide exchange in the alveoli and to a secondary inflammation-mediated immune response disorder.

In the testing indicators, the authors indicated that the alveolar lavage operation technique was in accordance with the Expert Consensus of the Chinese Medical Association Respiratory Branch Regarding Pulmonary Infectious Disease Bronchial Alveolar Lavage Pathogen and Specimen Collection [2].

A short information on the applied technique, especially how much volume of saline solution was used in the alveolar lavage operation is lacking.

We want to draw the attention to our previous published article on alveolar neopterin, PCT, and IL-6 in relation to serum levels and severity of lung injury in ARDS [3]. PCT levels in bronchoalveolar lavage fluid (BALF) were not different between ARDS and acute lung injury (ALI). Concentrations of PCT in serum were significantly higher in patients with ARDS than in patients with ALI. Levels of IL-6 in BALF and in serum were higher in ARDS than in ALI. BAL (routine protocol for microbiological culture with 100 mL of 0.9% saline solution sequentially instilled and suctioned in 20 mL portions) was performed in s subsegment of the right middle lobe of lung within 12 and 24 h after the onset of ALI/ARDS. We determined the concentrations of PCT and IL-6 in the lung epithelial lining fluid (ELF) by using the urea-dilution method for estimating the volume of ELF in the lavaged lung segment [4]. Upon evaluating the combined group of patients with ALI/ARDS, the levels of PCT in ELF were significantly lower than in serum, whereas the levels of IL-6 in ELF exceeded significantly than that in serum.

Our results are different from the results of the paper by Liao and Yang [1]. Concentrations of PCT and IL-6 were not statistically different between survivors and non-survivors, and only PCT in BALF (*p* = 0.0759) and IL-6 in serum (*i* = 0.0630) tended to be higher in non-survivors [3]. Tsantes et al. evaluated plasma and BALF levels of PCT and IL-6 in discriminating septic from non-septic causes of ARDS [5]. They concluded that early plasma, but non-BALF-PCT concentrations can help to distinguish between septic and non-septic ARDS causes and are associated with the severity of multiple organ dysfunction syndrome in septic patients.
